# Electromagnetic Tissue Properties Imaging for Biomedical Applications

**DOI:** 10.1155/2013/461846

**Published:** 2013-11-12

**Authors:** Jin Keun Seo, William Lionheart, Ulrich Katscher, EungJe Woo

**Affiliations:** ^1^Department of Computational Science & Engineering/Mathematics, Yonsei University, Seoul 120-749, Republic of Korea; ^2^School of Mathematics, University of Manchester, Alan Turing Building, Oxford Road, Manchester M13 9PL, UK; ^3^Philips Technologie GmbH, Roentgenstraße 24-26, Hamburg, Germany; ^4^Department of Biomedical Engineering, Kyung Hee University, Gyeonggi-do 446-701, Republic of Korea

Recently, imaging techniques in science, engineering, and medicine have evolved to expand our ability to visualize a property of an object such as the human body. In particular, there has been marked progress in electromagnetic property imaging techniques, where cross sectional image reconstructions of electric conductivity, permittivity, and magnetic susceptibility distributions inside the human body are pursued. These techniques also have wider application to imaging methods in medicine, biotechnology, nondestructive testing, monitoring of industrial process, and others.

This special issue focuses on imaging methodologies, mathematical models, and computational algorithms for imaging electrical tissue properties for biomedical applications. The imaging problems in this topic can be formulated as inverse problems that are intrinsically nonlinear. Finding solutions with practical significance and value requires in-depth understanding of the underlying physical phenomena and data acquisition systems as well as implementation details of image reconstruction algorithms. Experience over the last three decades has shown that the symbiotic interplay between theoretical mathematics, computational mathematics, and experiments is crucial for understanding and solving these nonlinear problems in practice.

 With this special issue, we hope to give an opportunity for this scientific community to consolidate knowledge in the field and to identify the new challenges and the most promising directions for future progress.

*Jin Keun Seo* *William Lionheart* *Ulrich Katscher* *EungJe Woo*

